# Supplementary material for: Impact of Integrated Genetic Information on Diagnosis and Prognostication for Myeloproliferative Neoplasms in the Next-Generation Sequencing Era
Source: J Clin Med. 2021 Mar 3;10(5):1033. doi: 10.3390/jcm10051033 (PMC7959293; doi:10.3390/jcm10051033)
Supplement: Supplementary file 1 [file jcm-10-01033-s001.pdf]

Supplementary Table S1. List of target genes in the NGS panel.

Target genes	Interval	Number of regions	Size (base pairs)
ABCA12	chr2:215797358-216002931	54	7929
ABL1	chr9:133589707-133761070	12	3529
ASXL1	chr20:30946579-31025141	17	4720
ATM	chr11:108098352-108236235	62	9171
ATRX	chrX:76763829-77041487	36	7543
ATXN7L1	chr7:105248299-105517004	15	3132
BCOR	chrX:39909169-39937182	15	5348
BRAF	chr7:140426294-140624503	21	2379
BRCC3	chrX:154299803-154348425	11	1021
CALR	chr19:13049494-13054795	10	1294
CBL	chr11:119077128-119170491	16	2721
CBLB	chr3:105377814-105588232	20	3079
CD101	chr1:117544440-117576723	9	3066
CEBPA	chr19:33792244-33793425	1	1182
CREBBP	chr16:3777719-3929917	31	7368
CSF1R	chr5:149433632-149465990	22	3003
CSF3R	chr1:36931697-36945097	16	2698
CTCF	chr16:67644736-67671775	10	2184
CUX1	chr7:101459311-101926382	34	5433
DNMT1	chr19:10244343-10311559	43	5292
DNMT3A	chr2:25457148-25536853	25	2888
EGFR	chr7:55086971-55273310	31	4084
EP300	chr22:41489009-41574960	31	7245
ERG	chr21:39739557-39947624	12	1764
ETV6	chr12:11803062-12044535	10	1443
EZH2	chr7:148504738-148544390	21	2456
FBXW7	chr4:153244033-153332955	14	2618
FLT3	chr13:28578189-28674647	25	3004
GATA1	chrX:48649517-48652675	5	1346
GATA2	chr3:128199862-128205874	5	1443
GNAS	chr20:57415162-57485884	17	4096
HIPK2	chr7:139257673-139477422	16	3708
IDH1	chr2:209101803-209116275	8	1248
IDH2	chr15:90627498-90645622	11	1359
INVS	chr9:102866804-103062956	17	3395
IRF1	chr5:131819643-131825170	9	978
JAK2	chr9:5021988-5126791	23	3399
KDM2B	chr12:121867919-122018816	29	4276
KDM6A	chrX:44732798-44970656	31	4470
KIT	chr4:55524182-55604723	21	2931
KMT2A	chr11:118307228-118392887	38	12082
KMT2D	chr12:49415563-49449107	55	16662
KRAS	chr12:25362729-25398318	6	708
LAMB4	chr7:107664484-107763609	35	5507
MECOM	chr3:168802697-169381160	20	3816
MET	chr7:116335811-116436178	21	4359
MLL3	chr7:151833917-152132871	62	15030
MLL5	chr7:104681400-104753780	27	5743
MN1	chr22:28146903-28196531	2	3963
MPL	chr1:43803520-43818443	12	1993
NCOR2	chr12:124809948-124979797	49	7734
NF1	chr17:29422226-29705949	63	9011
NLRP1	chr17:5405134-5487277	18	4493
NOTCH1	chr9:139390523-139440238	34	7668
NPM1	chr5:170814953-170837569	12	894
NRAS	chr1:115251156-115258781	4	570
NRD1	chr1:52254908-52344287	34	3696
NUP98	chr11:3692612-3803347	35	5567
OCA2	chr15:28000534-28327020	24	2573
PDGFRA	chr4:55106220-55161439	24	3450
PHF12	chr17:27233201-27278622	17	3519
PHF6	chrX:133511648-133559360	9	1207
PRPF40B	chr12:50017374-50037975	26	2682

PRPF8	chr17:1553953-1587865	42	7151
PTPN11	chr12:112856916-112942568	16	1822
RAD21	chr8:117859739-117878968	13	1896
RAD50	chr5:131893017-131978781	27	4211
RINT1	chr7:105172763-105207758	16	2417
ROBO1	chr3:78648063-79639061	34	5223
ROBO2	chr3:75986645-77695209	32	4862
RUNX1	chr21:36164432-36421196	11	1584
RUNX1T1	chr8:92972470-93115112	20	2350
SETBP1	chr18:42281312-42643663	6	4980
SF3A1	chr22:30730583-30752781	16	2382
SF3B1	chr2:198257027-198299723	27	4045
SMC1A	chrX:53407024-53449549	26	3882
SMC3	chr10:112327575-112364060	29	3654
SRSF2	chr17:74732243-74733242	2	666
STAG2	chrX:123156478-123234447	34	3861
TET1	chr10:70332096-70451571	11	6411
TET2	chr4:106111627-106197676	10	6165
TP53	chr17:7565257-7579912	14	1378
TP53BP1	chr15:43699581-43785241	31	6130
U2AF1	chr21:44513212-44527604	9	790
U2AF2	chr19:56166471-56185434	14	1541
WT1	chr11:32410604-32456891	11	1568
ZRSR2	chrX:15808619-15841365	12	1690

Supplementary Table S2. Univariate analysis of clinical and genetic factors for overall survival, leukemic transformation, and fibrosis progression.

Variables	Overall survival			Leukemic transformation			Fibrosis progression		
	P	HR	95% CI	*P*	HR	95% CI	*P*	HR	95% CI
**Demographics**									
Diagnosis[a]	**0.0015**	**13.0**	**2.7-63.5**	0.0677	3.5	0.9-13.5	0.2424	1.7	0.7-3.9
Age at diagnosis	**0.0234**	**1.1**	**1.0-1.1**	0.1953	1.0	1.0-1.1	0.1090	1.0	0.9-1.0
Splenomegaly	0.4385	1.7	0.5-5.9	0.2077	2.3	0.6-8.9	0.4211	1.4	0.6-3.5
Vascular event	0.3313	0.4	0.0-2.8	0.6042	0.7	0.1-3.2	0.9801	1.0	0.4-2.7
Bone marrow fibrosis	**0.0346**	**9.3**	**1.2-73.6**	0.2183	2.7	0.6-13.3			
PB blast proportion	0.0750	1.2	1.0-1.4	**<0.0001**	**1.5**	**1.3-1.8**	**0.0011**	**1.3**	**1.1-1.4**
Hemoglobin level	**0.0012**	**0.6**	**0.5-0.8**	0.0516	0.8	0.6-1.0	**0.0060**	**0.8**	**0.7-0.9**
White blood cell count	0.1201	1.0	1.0-1.0	**0.0186**	**0.7**	**0.6-1.0**	0.5532	1.0	1.0-1.1
Platelet count	**0.0192**	**1.0**	**1.0-1.0**	0.1613	1.0	1.0-1.0	0.4431	1.0	1.0-1.0
**Triple mutations**									
JAK2	**0.0403**	**0.1**	**0.0-0.9**	0.8913	0.9	0.2-3.4	0.7567	0.9	0.4-2.0
CALR	0.9085	1.2	0.3-4.6	0.4695	1.6	0.4-6.2	0.7756	1.1	0.5-2.7
MPL	0.2307	3.5	0.4-28.1	0.9667			0.1888	2.7	0.6-12.0
**Other mutations[b]**									
ASXL1	0.0964	3.2	0.8-12.2	0.9254	1.1	0.1-9.0	**0.0045**	**4.1**	**1.5-10.8**
TET2	0.2384	2.5	0.5-12.0	0.8795	1.2	0.1-9.4	0.0740	3.3	0.9-12.1
U2AF1	11.9690	4.6	0.6-37.3	0.9667			NA		
DNMT3A	0.9554			0.5456	1.9	0.2-16.1	0.9542		
RUNX1	0.0941	6.0	0.7-49.0	**0.0252**	**13.3**	**1.4-128.7**	0.9722		
SF3B1	0.9612			0.9709			**0.0084**	**8.4**	**1.7-40.6**
TP53	**<0.0001**	**33.7**	**9.4-120.8**	**0.0005**	**13.6**	**3.1-59.2**	0.6805	1.4	0.3-6.0
SRSF2	0.9718			0.9637			0.9594		
IDH1/2	0.9687			**0.0063**	**7.3**	**1.8-30.2**	**0.0208**	**5.9**	**1.3-26.4**
**No. mutations**									
1	0.9625								
2	0.9616						**0.0348**	**2.8**	**1.1-7.2**
>2	0.9592			0.0959	3.3	0.8-13.2	**0.0161**	**4.2**	**1.3-13.4**
**Abnormal karyotype[c]**									
20q-	**0.0027**	**9.0**	**2.1-37.9**	0.2406	3.7	0.4-33.4	**0.0045**	**9.5**	**2.0-45.2**
13q-	**0.0075**	**6.9**	**1.7-28.1**	0.9662	1.0	0.1-8.3	0.3353	2.8	0.3-22.4
-5/5q-	**0.0001**	**18.1**	**4.7-69.7**	**0.0279**	**5.2**	**1.2-22.7**	0.6969	0.8	0.2-2.7
-7/7q-	**<0.0001**	**44.2**	**11.6-168.3**	**0.0022**	**14.7**	**2.6-82.1**	0.9116	1.1	0.1-8.5
**Complex karyotype**	**<0.0001**	**27.0**	**7.1-102.4**	**0.0012**	**12.7**	**2.7-58.9**	0.8063	1.2	0.3-5.3
**No. abnormal karyotype**									
1	**0.0432**	**11.9**	**1.1-131.4**				**0.0382**	**3.4**	**1.1-10.7**
2	**0.0438**	**17.5**	**1.1-282.3**						
>2	**<0.0001**	**88.2**	**10.2-764.7**	**0.0012**	**12.7**	**2.7-58.9**			

Bold indicates significant values with $P < 0.05$; The fibrosis progression was analyzed in patients with polycythemia and essential thrombocythemia, excluding primary myelofibrosis. [a]Diagnosis refers to primary myelofibrosis for overall survival and leukemic transformation, and essential thrombocythemia for fibrosis progression. [b]Frequently detected mutations and cytogenetic abnormalities (5 or more times) were included. *HR* hazard ratio, *CI* confidence interval, *PB* peripheral blood, *No.*number.

Supplementary Table S3. *P* values from log rank tests for overall survival, leukemic transformation, and fibrosis progression in each disease category.

Variables	Overall survival	Leukemic transformation			Fibrosis progression		
	**ET**	**PMF**	**PV**	**ET**	**PMF**	**PV**	**ET**
**Triple mutations**							
JAK2	0.3678	0.2789	0.5583	0.9779	0.9306	0.4245	0.5136
CALR	0.7966	0.4426		0.5937	0.8573		0.5656
MPL	**0.0073**	0.6945	0.8728	0.7324	0.8586		0.3888
**Other mutations[a]**							
ASXL1	0.5344	0.3659	0.8728	0.4748	0.6402		**0.0109**
TET2	0.7257	0.1304	0.5773	0.6889	0.1262	0.1187	0.3663
U2AF1	-	0.7933			0.5708		
DNMT3A	0.7257	0.7969	0.6158	0.2352	0.9005	0.7844	0.1083
RUNX1	0.9028	0.1550	0.8728	0.8415	**0.0079**		0.8063
SF3B1	0.8618	0.5474		0.7728	0.7979		**0.0098**
TP53	**<0.0001**	**<0.0001**	**0.0454**	**0.0056**	**< 0.0001**	0.5505	0.9340
SRSF2	0.9028	0.6056	0.8728		0.8259		0.3437
IDH1/2	0.7434	0.6760	0.0696	**0.0050**	0.8586	**< 0.0001**	0.5126
**No. mutations**	0.1539	0.1980	0.5726	0.8836	0.3961	0.4366	0.2778
**Abnormal karyotype[a]**							
20q-	0.8955	0.1525	0.8575		0.5929	**0.0363**	**< 0.0001**
13q-	**<0.0001**	0.9829		0.8273	0.6896		0.4613
-5/5q-	**0.0001**	**<0.0001**	0.2087	0.5586	**0.0004**	0.8421	0.5101
-7/7q-	**<0.0001**	**0.0005**		**0.0150**	**0.0261**		0.9312
**Complex karyotype**	**<0.0001**	**<0.0001**	0.1658	**0.0150**	**0.0004**	0.7431	0.9312
**No. abnormal karyotype**	**<0.0001**	**<0.0001**	0.3646	0.0936	**0.0031**	0.8767	**0.0016**

Bold indicates significant values with $P < 0.05$; [a]Frequently detected mutations and cytogenetic abnormalities (5 or more times) were included. *PV* polycythemia vera, *ET* essential thrombocythemia, *PMF* primary myelofibrosis, *No.* number.

Supplementary Table S4. Comparison of the prediction power of conventional risk groups and mutation and/or karyotype enhanced systems.

	Overall survival		Event free survival	
	**χ^2 value**	*P* value	χ^2 value	*P* value
**PV**				
Conventional risk group*			0.9	0.635
MIPSS-ET			0.4	0.815
**ET**				
Conventional risk group*	0.2	0.922	1.0	0.611
MIPSS-ET	**11.9**	**0.003**	4.2	0.120
**PMF**				
DIPSS	**9.4**	**0.024**	4.6	0.201
MIPSS70	**10.4**	**0.006**	**10.6**	**0.005**
MIPSS70+	**16.21**	**0.003**	**16.8**	**0.002**

The χ^2 and *P* values were derived from log-rank test comparisons of survival curves. * Conventional risk groups are described in Table 4. *PV* polycythemia vera, *ET* essential thrombocythemia, *PMF* primary myelofibrosis.

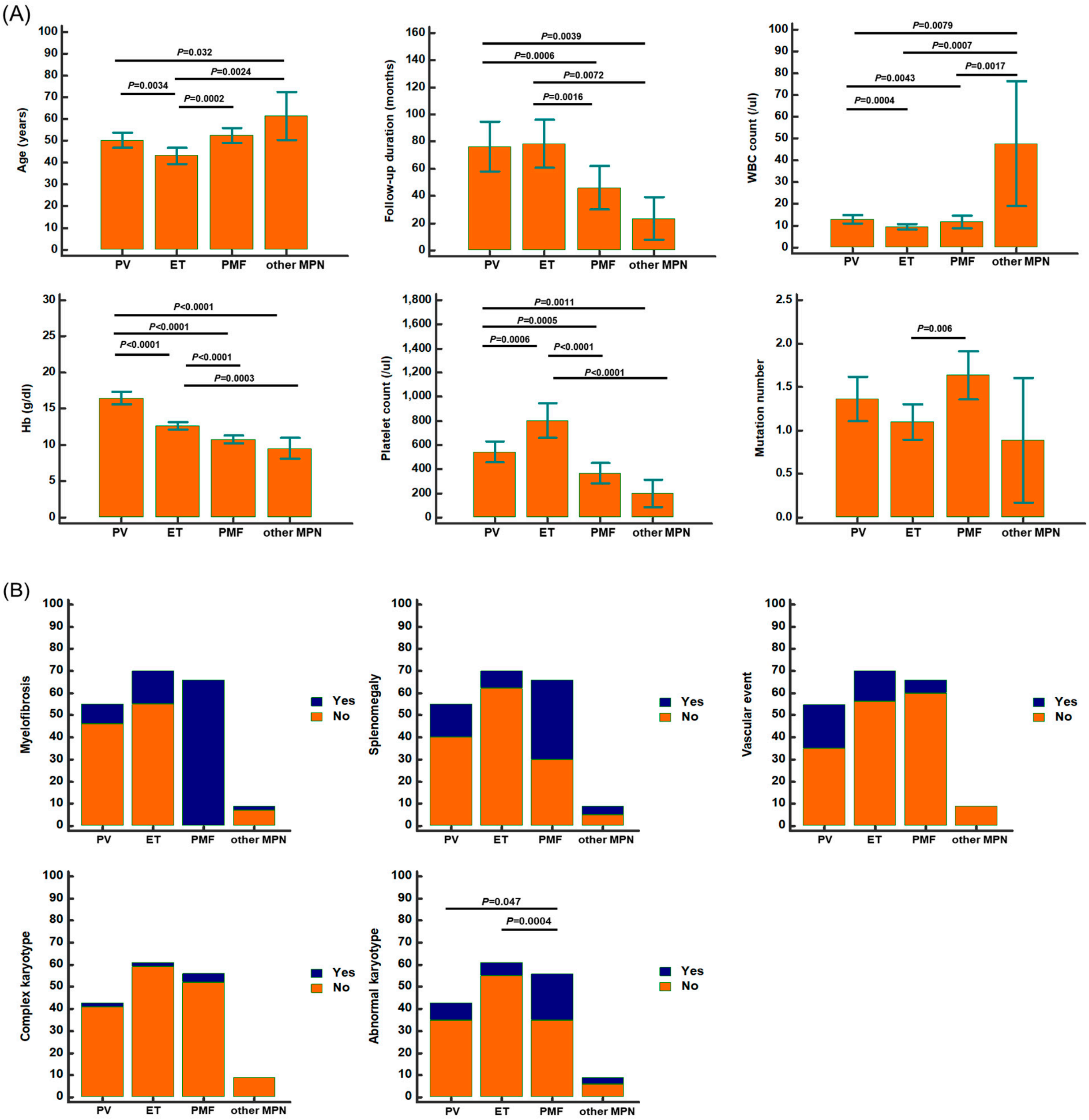

Supplementary Figure S1. Comparison of clinical and genetic variables among disease categories. (A) Bar charts represent mean and standard deviation of each numerical variable with *P* values analyzed by Mann-Whitney test. (B) Bar charts represent frequency of each variable with *P* values analyzed by Fisher's exact test.
